# Real-World Impact of Surgical Excision on Overall Survival in Primary Central Nervous System Lymphoma

**DOI:** 10.3389/fonc.2020.00131

**Published:** 2020-02-26

**Authors:** Xiangyang Deng, Xingxing Xu, Dongdong Lin, Xiaojia Zhang, Lisheng Yu, Hansong Sheng, Bo Yin, Nu Zhang, Jian Lin

**Affiliations:** ^1^Department of Neurosurgery, The Second Affiliated Hospital and Yuying Children's Hospital of Wenzhou Medical University, Wenzhou, China; ^2^School of Basic Medical Sciences, Wenzhou Medical University, Wenzhou, China

**Keywords:** surgery, primary central nervous system lymphoma, SEER, survival, nomogram

## Abstract

Substantial controversy still exists regarding the use of surgical excision in the treatment of primary central nervous system lymphoma (PCNSL). This study was aimed to evaluate the survival benefit of surgical excision in PCNSL patients based on a US population. Using the Surveillance, Epidemiology, and End Results (SEER) Program database, a total of 3,543 PCNSL patients were identified from 2000 to 2014 for analysis. Surgical excision was accessed via Kaplan–Meier and multivariate Cox regression survival analyses. Coarsened exact matching (CEM) analysis was additionally employed to intensify our findings. Finally, we found that surgical excision was significantly associated with increased survival over no surgery/biopsy (*P* < 0.001), and its survival benefit was also independent of baseline prognostic factors. The survival benefit of surgery was also validated in clinically important subsets. CEM analysis further validated the survival advantage of surgery (*P* < 0.001). Moreover, a novel prediction model with excellent performance was established to estimate the potential benefit from surgical excision of the lesion with respect to the end point of overall survival. The current study supports the favorable impact of surgical excision on clinical outcome in patients with PCNSL. Although further randomized controlled trials are warranted, currently available evidence should be considered in the clinical management of this disease.

## Introduction

Primary central nervous system lymphoma (PCNSL) is a rare and devastating extranodal non-Hodgkin lymphoma confined to the CNS, accounting for ~4% of all intracranial tumors (1–3). Incidence rates for PCNSL have been increased during recent decades with an annual incidence of 0.48/100,000 per year (4). PCNSL has traditionally carried a sinister prognosis, and 5-years survival is only 15–30% for these patients (2, 5). Despite that improved long-term survival has been reported for this tumor due to the substantial prognosis in therapeutic strategy (6–8), the overall prognosis remains frustrating, suggesting that intensive study of PCNSL is needed.

Stereotactic needle biopsy for diagnosis followed by systemic high-dose methotrexate-based chemotherapy is the current management paradigm for patients with PCNSL (9, 10). Considering exquisite sensitivity to chemotherapy and the risk of postoperative morbidity in these patients, surgery is reported to play a limited role in PCNSL compared to the management of other intracranial tumors, like diffuse gliomas (11–14). However, the widely adopted opinion of discouragement of surgery is based on some out-of-date retrospective studies with small samples (15–21). The absence of surgical effectiveness in these studies might attribute to the lack of in-depth understanding of this disease, the imperfection of treatment strategy, and the backwardness of neurosurgical techniques. Thus, in the era of modern neurosurgery, further evaluation of the role of surgical excision for PCNSL is needed.

In this study, we used population-based data to investigate the association of surgery with PCNSL prognosis to assess its efficacy. Coarsened exact matching (CEM) analysis, which is applied in oncology to assess treatment efficacies with the aim of minimizing selection bias (22), was additionally employed to intensify our findings. Furthermore, we developed a practical clinical tool for individualized survival prediction and estimation of potential benefit from surgical excision of the lesion with respect to the end point of overall survival (OS).

## Materials and Methods

### Data and Cohort Definition

The Surveillance, Epidemiology, and End Results (SEER) database, which collects patient-level demographics, lesion, and survival information from state cancer registries in the United States, was employed for this study. For our purposes, PCNSL patients were identified according to the International Classification of Diseases for Oncology Third Edition (ICD-O-3) histology codes (9590–9599, 9670–9699, 9700–9719, 9720–9729) and primary anatomic location (C71.0–C71.9). We restricted analysis to PCNSLs that were the first or only cancer, and those cases diagnosed without histological confirmation or diagnosed by autopsy were excluded. Patients with unknown survival information or surgery treatment were also excluded.

### Definition of Variables

The patient demographics including age at diagnosis (<50, 50–59, 60–69, 70–79, or ≥80 years), gender (male or female), race [white, black, American Indian/Alaska Native, or Asian Pacific Islander (AIAN/API), or unknown], and marital status (single, married, or unknown) were extracted from the SEER database. Tumor characteristics (tumor histological type and tumor site), treatment information (surgery, radiotherapy, and chemotherapy) and survival data were also acquired. Tumor histological type was grouped as diffuse large B-cell lymphoma (DLBCL), non-diffuse large B-cell lymphoma (non-DLBCL), or not-otherwise-specified (NOS) lymphoma. Tumor location was divided into supratentorial, infratentorial, or others/brain, NOS. According to SEER site-specific coding guidelines, the surgery treatment was categorized as no surgery/biopsy, subtotal resection (STR), and gross total resection (GTR).

### Statistical Analysis

The clinical end point for analysis was OS, defined as the length of time in months from diagnosis to death from any cause or last follow-up. Data were first described using summary statistics. Categorical variables between no surgery/biopsy and receipt of surgical excision (STR/GTR) were assessed with chi-square tests. Survival curves were depicted via Kaplan–Meier method and assessed by log-rank tests. Multivariate Cox analysis was applied to estimate the effect of covariates of interest on OS and identify independent prognostic factors.

Considering the potential selection, CEM, which is able to achieve lower levels of imbalance, model dependence, and bias than propensity score matching (23), was used to obtain a matched cohort for further evaluation of the role of surgical intervention in PCNSL.

Moreover, a nomogram model was established to predict the 1-, 3-, and 5-years OS for PCNSL patients, given the related risk factors. Calibration curves were used to access the consistency between nomogram-predicted survival and observed outcome, and the predictions should fall on a 45-degree diagonal line in a well-calibrated model. Concordance index (C-index) and time-dependent receiver operating characteristic curve (ROC) with the area under the curve (AUC) value were utilized to evaluate the discrimination of the nomogram model (24, 25). Bootstrap analyses with 1,000 resamples were conducted for these analyses. Statistical tests were two-sided, and *P* < 0.05 was considered statistically significant. All statistical analyses were performed using R version 3.2.3 software.

## Results

### Patient Characteristics

After applying the inclusion and exclusion criteria, a total of 3,543 patients were included in our study. Patient demographics, tumor characteristics, and treatment information are summarized in Table 1. In this cohort, 21.8% of patients were age <50 years, 18.6% were age 50–59 years, 26.1% were age 60–69 years, 24.1% were age 70–79 years, and only 9.3% were age ≥80 years. More than half (53.5%) of the patients were male, and white (79.7%) accounted for the majority. DLBCL (77.2%) was the most prevalent non-Hodgkin's lymphoma, and most tumors were located in a supratentorial location (51.2%). Additionally, the majority of patients received chemotherapy, whereas only 24.1% of patients received surgical excision.

**Table 1:** Patient demographics and disease characteristics.

**Parameters**	**Total patients**	**No surgery**	**Surgical excision**	P^*^
Entire cohort	3,543(100%)	2,692 (100%)	851 (100%)	
Age, years				0.371
<50	774 (21.8%)	595 (22.1%)	179 (21.0%)	
50–59	660 (18.6%)	493 (18.3%)	167 (19.6%)	
60–69	924 (26.1%)	685 (25.4%)	239 (28.1%)	
70–79	855 (24.1%)	663 (24.6%)	192 (22.6%)	
≥80	330 (9.3%)	256 (9.5%)	74 (8.7%)	
Sex				0.011
Male	1,895 (53.5%)	1,472 (54.7%)	423 (49.7%)	
Female	1,648 (46.5%)	1,220 (45.3%)	428 (50.3%)	
Race				0.676
White	2,822 (79.7%)	2,135 (79.3%)	687 (80.7%)	
Black	292 (8.2%)	230 (8.5%)	62 (7.3%)	
AIAN/API	418 (11.8%)	319 (11.8%)	99 (11.6%)	
Unknown	11 (0.3%)	8 (0.3%)	3 (0.4%)	
Year of diagnosis				0.006
2000–2007	1,790 (50.5%)	1,325 (49.2%)	465 (54.6%)	
2008–2014	1,753 (49.5%)	1,367 (50.8%)	386 (45.4%)	
Histological type				<0.001
DLBCL	2,736 (77.2%)	2,085 (77.5%)	651 (76.5%)	
Non-DLBCL	266 (7.5%)	161 (6.0%)	105 (12.3%)	
Lymphoma, NOS	541 (15.3%)	446 (16.6%)	95 (11.2%)	
Tumor site				<0.001
Supratentorial	1,814 (51.2%)	1,303 (48.4%)	511 (60.0%)	
Infratentorial	254 (7.2%)	151 (5.6%)	103 (12.1%)	
Other/brain, NOS	1,475 (41.6%)	1,238 (46.0%)	237 (27.8%)	
Surgery treatment				
No surgery/biopsy	2,692 (76.0%)	2,692 (100%)		
STR	424 (12.0%)		424 (49.8%)	
GTR	427 (12.1%)		427 (50.2%)	
Radiotherapy				0.574
No/unknown	2,285 (64.5%)	1,743 (64.7%)	542 (63.7%)	
Yes	1,258 (35.5%)	949 (35.3%)	309 (36.3%)	
Chemotherapy				0.428
No/unknown	1,281 (36.2%)	983 (36.5%)	298 (35.0%)	
Yes	2,262 (63.8%)	1,709 (63.5%)	553 (65.0%)	
Marital status				0.369
Single	1,432 (40.4%)	1,102 (40.9%)	330 (38.8%)	
Married	1,994 (56.3%)	1,498 (55.6%)	496 (58.3%)	
Unknown	117 (3.3%)	92 (3.4%)	25 (2.9%)	

*AIAN/API, American Indian/Alaska Native or Asian Pacific Islander; DLBCL, diffuse large B-cell lymphoma; NOS, not otherwise specified; STR, subtotal resection; GTR, gross total resection*.

* *P-value from chi-square tests*.

Furthermore, we observed that female patients and supratentorial tumors were more likely to undergo surgical resection.

### Association of Surgical Excision With Overall Survival

**Figure 1:**
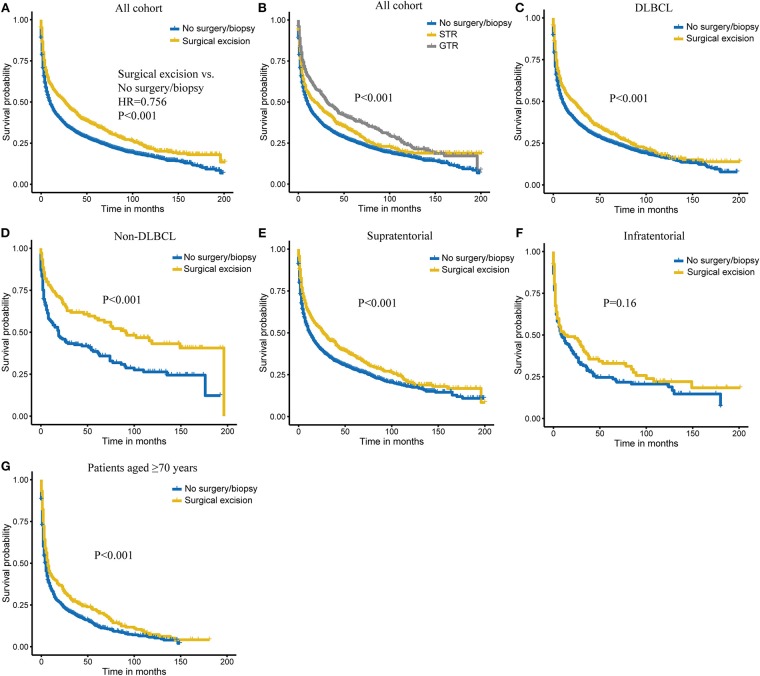
Kaplan–Meier survival of surgery **(A)** and extent of surgery **(B)** in all cohorts. Kaplan–Meier survival of surgical excision vs. no surgery/biopsy in diffuse large B-cell lymphoma (DLBCL) **(C)**, non-diffuse large B-cell lymphoma (non-DLBCL) **(D)**, supratentorial lymphoma **(E)**, infratentorial lymphoma **(F)**, and patients aged ≥70 years **(G)** subgroups.

Overall, 1-, 3-, and 5-years probabilities of survival in the entire cohort were 49.8% (95% CI, 48.2–51.4%), 35.5% (95% CI, 33.9–37.1%), and 28.6% (95% CI, 27.0–30.2%). A total of 2,262 patients (24.1%) received chemotherapy, yielding 1-, 3-, and 5-years Kaplan–Meier OS estimates of 63.6% (95% CI, 61.6–65.6%), 46.7% (95% CI, 44.5–48.9%), and 37.3% (95% CI, 35.1–39.5%), respectively. On univariate analysis, age at diagnosis, race, tumor histological type, tumor site, surgery treatment, radiotherapy, chemotherapy, and married status were significantly associated with OS (all *P* < 0.05). The 1-, 3-, and 5-years OS rate were 59.2, 44.7, and 36.0% for patients who underwent surgical excision, and 46.8, 32.5, and 26.3% for patients who received no surgery/biopsy (Table 2, Figure 1A; *P* < 0.001). Also, we additionally explored the extent of surgery on PCNSL outcome and found that GTR was associated with a survival benefit over STR (Figure 1B; *P* < 0.001). On multivariate Cox analysis, age at diagnosis, gender, year of diagnosis, tumor histological type, chemotherapy, and marital status were independent prognostic factors, while race had a borderline significance (Table 3). Surgical excision was significantly in correlation with an additive increase on survival [GTR vs. No surgery/biopsy: hazard ratio (HR) 0.69, *P* < 0.001; STR vs. No surgery/biopsy: 0.87, *P* = 0.028].

**Table 2:** OS at 1, 3, and 5 years.

	**1 year**		**3 years**		**5 years**		
**Parameter**	**OS**	**95% CI**	**OS**	**95% CI**	**OS**	**95% CI**	P^*^
Entire cohort	49.8	48.2–51.4	35.5	33.9–37.1	28.6	27.0–30.2	
Age, years							<0.001
<50	58.1	54.6–61.6	47.5	44.0–51.0	42.6	39.1–46.1	
50–59	62.0	58.3–65.7	46.1	42.2–50.0	36.9	33.0–40.8	
60–69	53.3	50.0–56.4	36.3	33.2–39.4	28.8	25.7–31.9	
70–79	38.7	35.4–42.0	24.3	21.4–27.2	17.3	14.6–20.0	
≥80	25.0	20.3–29.7	13.0	9.3–16.7	7.6	4.7–10.5	
Sex							0.443
Male	49.3	46.9–51.7	34.7	32.5–36.9	28.2	26.0–30.4	
Female	50.3	47.9–52.7	36.3	33.9–38.7	29.1	26.7–31.5	
Race							<0.001
White	49.6	47.8–51.4	34.8	33.0–36.6	27.6	25.8–29.4	
Black	39.2	33.5–44.9	31.5	26.2–36.8	28.0	22.7–33.3	
AIAN/API	57.3	52.6–62.0	41.7	36.8–46.8	34.5	29.6–39.4	
Unknown	~	~	~	~	~	~	
Histological type							<0.001
DLBCL	49.6	47.64–51.6	35.0	33.2–36.8	27.5	25.7–29.3	
Non-DLBCL	63.4	57.5–69.3	50.2	44.1–56.3	46.5	40.4–52.6	
Lymphoma, NOS	43.7	39.4–48.0	30.7	26.8–34.6	25.3	21.6–29.0	
Tumor site							0.005
Supratentorial	52.8	50.4–55.2	37.6	35.2–40.0	30.4	28.2–32.6	
Infratentorial	47.8	41.7–53.9	32.8	26.9–38.7	28.0	22.3–33.7	
Other/brain, NOS	46.4	43.9–48.9	33.3	30.9–35.7	26.5	24.1–28.9	
Surgery treatment							<0.001
No surgery/biopsy	46.8	44.8–48.8	32.5	30.7–34.3	26.3	24.5–28.1	
STR	53.9	49.2–58.6	41.3	36.6–46.0	32.3	27.6–37.0	
GTR	64.4	59.9–68.9	48.0	43.1–52.9	39.7	34.8–44.6	
Radiotherapy							0.010
No/unknown	49.7	47.5–51.9	38.2	36.2–40.2	32.2	30.2–34.2	
Yes	50.0	47.3–52.7	30.6	28.1–33.1	22.6	20.2–25.0	
Chemotherapy							<0.001
No/unknown	25.1	22.7–27.5	15.4	13.4–17.4	13.1	11.1–15.1	
Yes	63.6	61.6–65.6	46.7	44.5–48.9	37.3	35.1–39.5	
Marital status							<0.001
Single	45.2	42.7–47.7	31.1	28.7–33.5	25.3	22.9–27.7	
Married	53.6	51.4–55.8	39.0	36.8–41.2	31.4	29.2–33.6	
Unknown	39.7	30.9–48.5	29.3	21.1–37.5	22.9	14.7–31.1	

*OS, overall survival; AIAN/API, American Indian/Alaska Native or Asian Pacific Islander; DLBCL, diffuse large B-cell lymphoma; NOS, not otherwise specified; STR, subtotal resection; GTR, gross total resection*.

* *P-value from log-rank test*.

**Table 3:** Multivariate cox analysis.

**Parameters**	**Hazard ratio**	**95% CI**	***P***
**Age, years**			
<50	Reference		
50–59	1.37	1.20–1.57	<0.001
60–69	1.75	1.55–1.98	<0.001
70–79	2.15	1.90–2.44	<0.001
≥80	2.69	2.31–3.13	<0.001
**Gender**			
Male	Reference		
Female	0.89	0.821–0.961	0.003
**Race**			
White	Reference		
Black	1.14	0.99–1.33	0.074
AIAN/API	0.90	0.80–1.02	0.110
Unknown	~	~	~
**Year of diagnosis (continued)**	0.98	0.97–0.99	<0.001
**Histological type**			
DLBCL	Reference		
Non-DLBCL	0.60	0.51–0.70	<0.001
Lymphoma, NOS	0.93	0.83–1.03	0.151
**Tumor site**			
Supratentorial	Reference		
Infratentorial	1.14	0.98–1.32	0.098
Others/brain, NOS	1.17	1.08–1.26	<0.001
**Surgery treatment**			
No surgery/biopsy	Reference		
STR	0.87	0.77–0.99	0.028
GTR	0.69	0.61–0.78	<0.001
**Radiotherapy**			
No/unknown	Reference		
Yes	0.94	0.87–1.02	0.147
**Chemotherapy**			
No/unknown	Reference		
Yes	0.43	0.39–0.47	<0.001
**Marital status**			
Single	Reference		
Married	0.83	0.77–0.90	<0.001
Unknown	0.94	0.76–1.17	0.583

*AIAN/API, American Indian/Alaska Native or Asian Pacific Islander; DLBCL, diffuse large B-cell lymphoma; NOS, not otherwise specified; STR, subtotal resection; GTR, gross total resection*.

### Stratification Analyses

To determine whether the survival benefit of surgical resection is robust in different subgroups, stratification analyses were carried out and showed that surgical excision achieved better survival in DLBCL group, non-DLBCL group, and patients with supratentorial tumors (all *P* < 0.001; Figures 1C–E). We also observed a similar trend with survival for surgical excision in infratentorial tumors, although without reaching a statistical significance (Figure 1F). Considering that older patients are at higher risk for operative complications and postoperative death, we further evaluated the role of surgery in older patients (≥70 years). As shown in Figure 1G, these patients could also benefit from surgical intervention (*P* < 0.001).

### Combined Effect of Surgery and Chemotherapy on Survival

**Figure 2:**
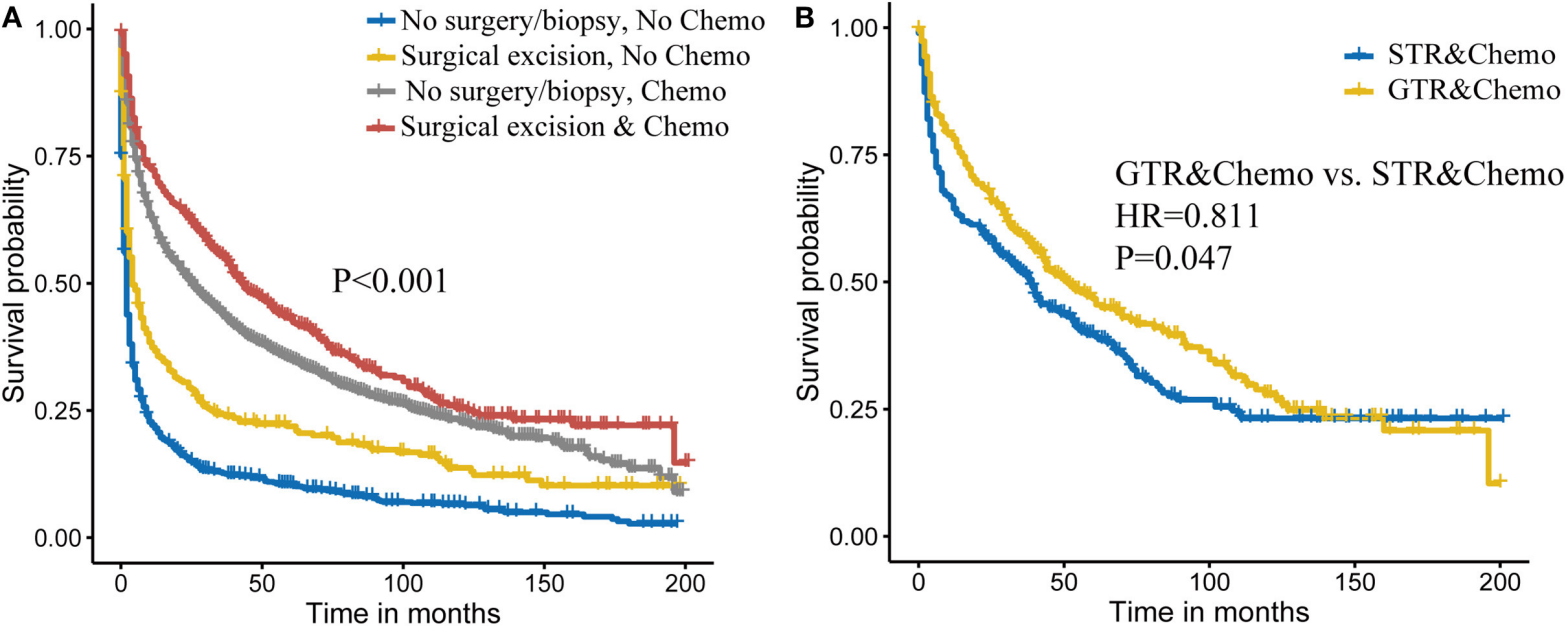
Combined effect of surgery and chemotherapy on primary central nervous system lymphoma (PCNSL) survival **(A)**. Extent of the surgery and chemotherapy on PCNSL survival **(B)**.

Then, we attempted to explore the combined effect of surgery and chemotherapy on PCNSL outcome. As shown in Figure 2A, we found that combining surgical excision and chemotherapy was related to best outcome. For specific surgery types, combining GTR and chemotherapy achieved better survival (HR = 0.811, *P* = 0.047; Figure 2B).

### Statistical Matching for Surgical Excision

**Figure 3:**
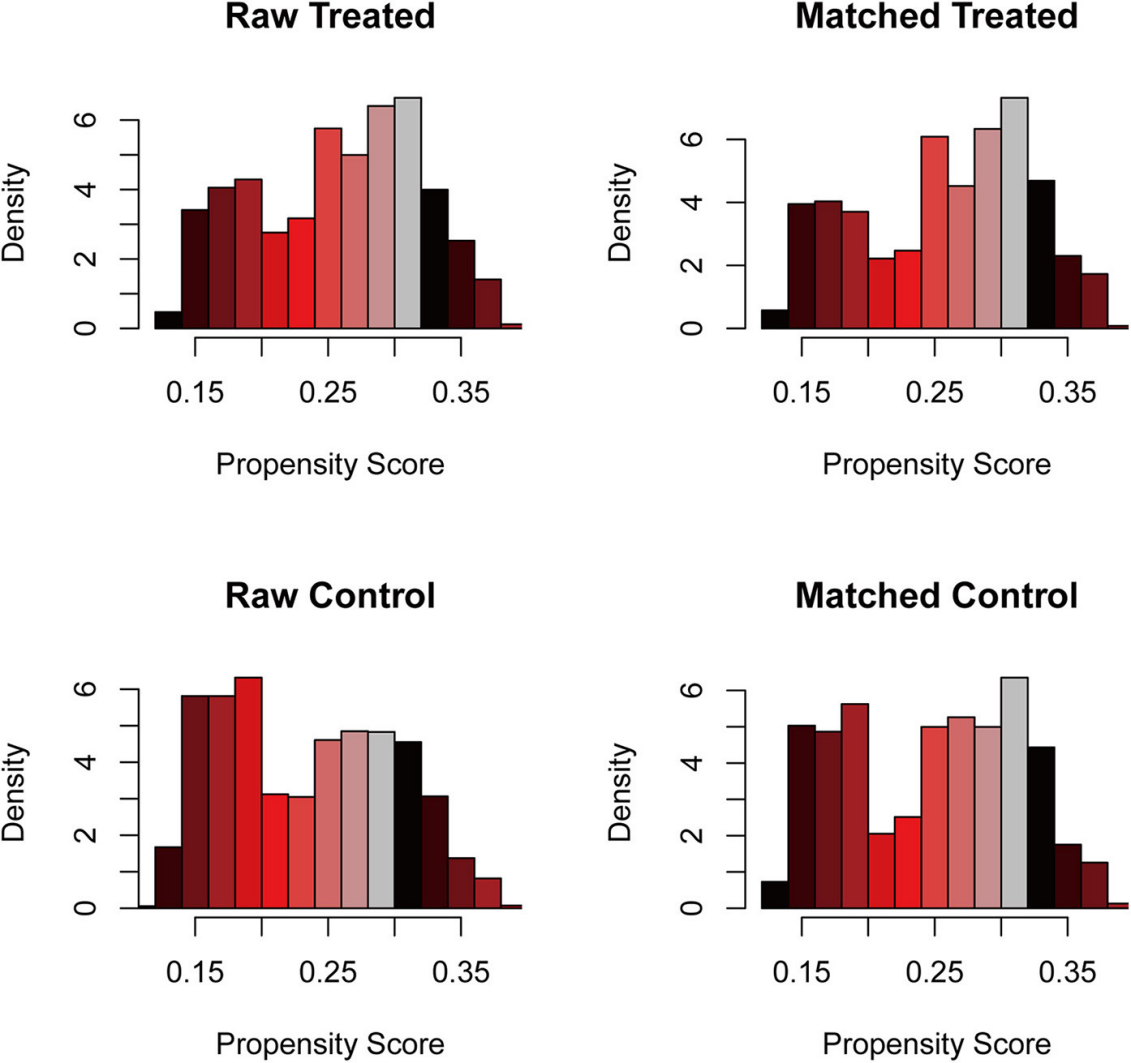
The histogram of raw data and matched data for surgical excision. The histograms before matching were on the left, while the histograms after matching were on the right. The similarity between treated and control group was correlated with the success of matching.

**Figure 4:**
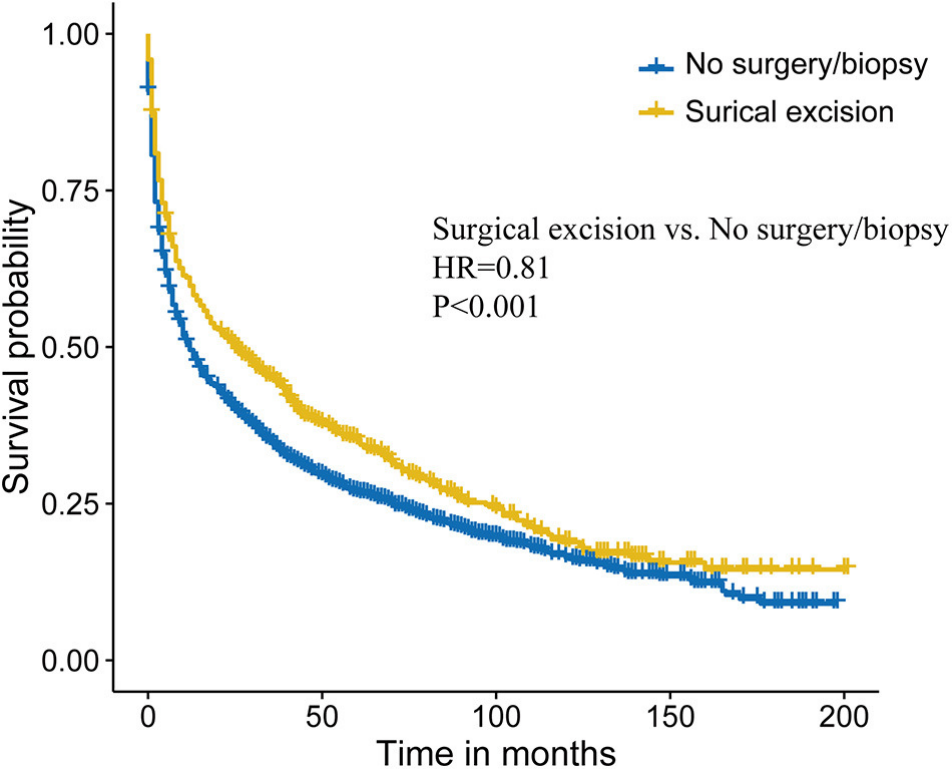
Survival analysis comparing surgical excision vs. no surgery/biopsy after coarsened exact matching (CEM) in primary central nervous system lymphoma (PCNSL).

To further intensify our findings, we additionally performed CEM analysis for surgery treatment to access the role of surgical excision in PCNSL. The mean difference between no surgery/biopsy group and surgical excision group of all included variables was decreased via matching. The histograms after CEM (right side ones) were much more similar than the left side ones without CEM (Figure 3), indicating that potential selection bias associated with the receipt of surgery treatment was minimized. Then, the Kaplan–Meier analysis was conducted for the new matched data, and we found that surgical excision still conferred a survival advantage (HR, 0.81, *P* < 0.001; Figure 4).

### Nomogram Development and Internal Validation

**Figure 5:**
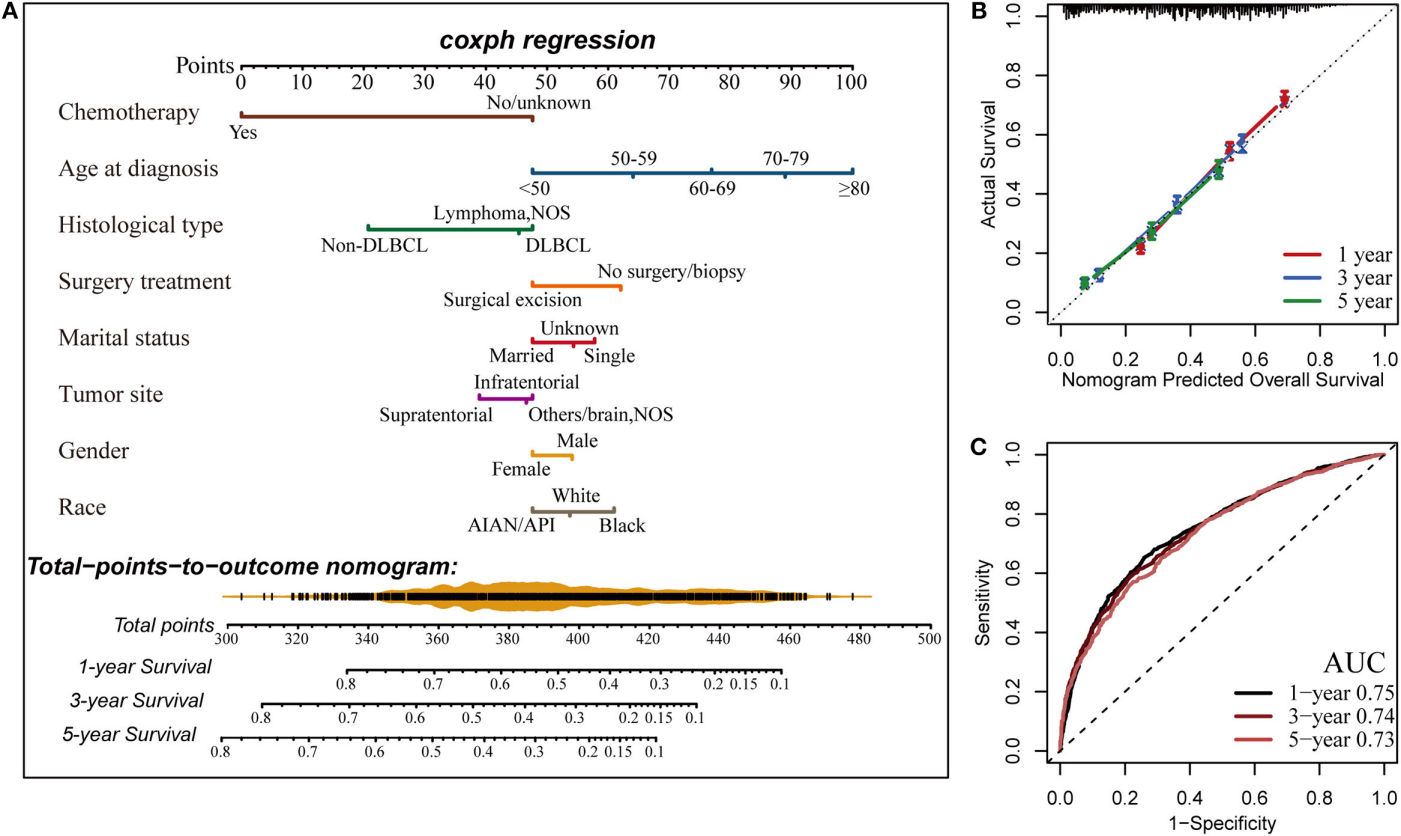
Construction of the nomogram for estimating the probability of 1-, 3-, and 5-years overall survival for primary central nervous system lymphoma (PCNSL) **(A)**. Calibration plot of the nomogram for predicting the probability of overall survival at 1, 3, and 5 years **(B)**. The time-dependent receiver operating characteristic curve (ROC) analysis showed that the nomogram had the best performance **(C)**. AUC, area under the curve.

Finally, a nomogram was developed to predict 1-, 3-, and 5-years OS for PCNSL patients on the basis of the results of multivariate Cox analysis (Figure 5A). The nomogram model was internally validated by bootstrap validation method. This model demonstrated favorable discrimination with an unadjusted C-index of 0.688 and a bootstrap-corrected C-index of 0.688. Calibration curves exhibited excellent concordance between the nomogram-predicted survival and actual outcome (Figure 5B). The ROC analysis also indicated that the nomogram model had favorable prognostic accuracy of OS (1-year AUC, 0.75; 3-years AUC, 0.74; 5-years AUC, 0.73; Figure 5C).

## Discussion

Surgery is discouraged for PCNSL according to the current treatment paradigm which is based on older studies. Although several recent studies have attempted to clarify the impact of surgical excision on PCNSL survival and achieved positive results, substantial controversy still exists regarding the use of surgery in the treatment of these tumors. Based on the study cohort extracted from SEER database, we found that surgical excision was significantly associated with increased survival over no surgery/biopsy, and its survival benefit was also independent of baseline prognostic factors, as well as chemotherapy after multivariate Cox analysis.

Then, we explored the combined effect of surgery and chemotherapy and found that combining surgical excision and chemotherapy conferred a better outcome over chemotherapy alone, suggesting that multimodality treatment might be more beneficial. For the specific extent of excision, more extensive resection was observed to lead to a better survival. Moreover, considering the heterogeneity of clinical outcomes of the PCNSL, we also validated the survival benefit of surgery in clinically important subsets.

Several uncontrolled retrospective studies have found surgery to have a favorable impact on the PCNSL outcome. Weller et al. (26) did a secondary analysis of the German PCNSL Study Group-1 trial, which was designed to examine the role of brain radiotherapy in the treatment of PCNSL and first demonstrated the superiority of surgical resection over biopsy on PCNSL progression-free survival and OS. Jelicic et al. (27) reported the benefit of radical surgical approach in their retrospective study of 27 cases of PCNSL, but their small sample size might make their conclusions unreliable. The study of Rae et al. (10) had also come to a similar conclusion, but it was subject to selection bias as well. Tumor debulking could contribute to a decrease in intracranial pressure for patients with a large lesion to improve neurological symptoms and tolerance for upcoming intensive chemotherapy (2, 10), combined with improved safety of surgery due to advances in neurosurgical techniques, and might yield good results for PCNSL patients. Consistently, our study also provided evidence of a clear association of surgical excision in PCNSL patients with increased survival. Given the retrospective nature, the potential selection bias cannot be precluded via usual multivariable adjustment, we additionally applied CEM analysis to further strengthen the credibility of our conclusion.

Moreover, in addition to providing estimates of baseline probability of OS, our newly built nomogram model also provides an individualized quantitative potential benefit from surgical excision for PCNSL patients. For example, a 66-year-old (77 points) white (54 points) married (48 points) man (54 points) had a supratentorial (39 points) lymphoma with unknown concrete subtype (45 points), who intends to undergo surgical resection (48 points) followed by chemotherapy (0 points), gets a total of 365 points, yielding an estimated 3-years OS of 53%. However, the estimated 3-years OS rate would only be 43% if this patient did not receive surgical excision of the lesion, suggesting an obvious benefit from surgery for this patient. Thus, this practical clinical tool could provide more distinct and direct data to assist in clinical decision making and optimization of therapeutic approaches in clinical care.

Investigation of nationwide datasets is of high value in rare diseases, like PCNSL, and has been advocated (28). Although we clearly demonstrated the benefit of surgery for PCNSL patients and provided a quantifiable tool, several limitations should be acknowledged in our study. First, the bias attributing to the imbalance between surgical excision group and no surgery/biopsy group could not be eliminated. However, both multivariable Cox analysis and additional CEM analysis were employed to reduce potential confounding, making our results more convincing. Secondly, unidentified factors including details of the lesion, patient performance status, clinical symptoms, comorbidities, recurrence status, and the type of radiotherapy and chemotherapy were not adjusted due to the inherent limitation of the SEER database. Thirdly, the lack of validation of the SEER data for included variables could raise some concern about our study, but these registry data usually provide a large sample size with high completeness and representativeness, making the influence of this deficiency on the results decreased to some extent. Finally, our study laid the foundation for the establishment of the survival prediction model in PCNSL, but this model is yet to be improved via integrating more clinicopathologic parameters and externally validated.

In summary, this is the first study to use CEM analysis for analyzing surgical excision in PCNSL and establish a novel prediction model for these patients. The current study supports the favorable impact of surgery on clinical outcome in patients with PCNSL. Although further randomized controlled trials is warranted, currently available evidence should be considered in the clinical management of this disease.

## Data Availability Statement

Publicly available datasets were analyzed in this study. This data can be found here: https://seer.cancer.gov/data/.

## Author Contributions

XD and JL designed the study. XD, XX, DL, and XZ contributed to data analysis. XD and XX wrote the initial draft of the manuscript. LY, HS, BY, NZ, and JL reviewed and edited the manuscript. All authors read and approved the manuscript.

### Conflict of Interest

The authors declare that the research was conducted in the absence of any commercial or financial relationships that could be construed as a potential conflict of interest.
